# 719 Decreased Morbidity in Patients with Hidradenitis Suppurativa Insured by Medicaid or Medicare

**DOI:** 10.1093/jbcr/irad045.194

**Published:** 2023-05-15

**Authors:** Rena Atayeva, Rafael Felix Tiongco, Julie Caffrey, Carisa Cooney, Jonathan Lee

**Affiliations:** Johns Hopkins University School of Medicine, Baltimore, Maryland; Johns Hopkins University School of Medicine, Baltimore, Maryland; Johns Hopkins University School of Medicine, Baltimore, Maryland; Johns Hopkins University School of Medicine, Baltimore, Maryland; Johns Hopkins University, Baltimore, Maryland; Johns Hopkins University School of Medicine, Baltimore, Maryland; Johns Hopkins University School of Medicine, Baltimore, Maryland; Johns Hopkins University School of Medicine, Baltimore, Maryland; Johns Hopkins University School of Medicine, Baltimore, Maryland; Johns Hopkins University, Baltimore, Maryland; Johns Hopkins University School of Medicine, Baltimore, Maryland; Johns Hopkins University School of Medicine, Baltimore, Maryland; Johns Hopkins University School of Medicine, Baltimore, Maryland; Johns Hopkins University School of Medicine, Baltimore, Maryland; Johns Hopkins University, Baltimore, Maryland; Johns Hopkins University School of Medicine, Baltimore, Maryland; Johns Hopkins University School of Medicine, Baltimore, Maryland; Johns Hopkins University School of Medicine, Baltimore, Maryland; Johns Hopkins University School of Medicine, Baltimore, Maryland; Johns Hopkins University, Baltimore, Maryland; Johns Hopkins University School of Medicine, Baltimore, Maryland; Johns Hopkins University School of Medicine, Baltimore, Maryland; Johns Hopkins University School of Medicine, Baltimore, Maryland; Johns Hopkins University School of Medicine, Baltimore, Maryland; Johns Hopkins University, Baltimore, Maryland

## Abstract

**Introduction:**

Hidradenitis suppurativa (HS) is a chronic condition that is defined by recurring inflammation and fibrosis of intertriginous areas. Patients with HS have had increasing inpatient hospitalization rates over the years. Our study investigates inpatient mortality and morbidity factors in patients with HS by primary payer status. We hypothesized that patients who are uninsured or insured by Medicaid or Medicare will have worse in-hospital mortality rates when compared to those insured by private insurance.

**Methods:**

Using a national database, we performed a cross-sectional study of patients hospitalized from 2017-2019 with a primary diagnosis of HS. The sample size included 12,365 patients with HS. The independent variable was primary payer status, and the dependent variables were age, gender, race, and zip code income quartiles. The primary outcome was in-hospital mortality. The secondary outcomes were morbidity factors (infection and wound dehiscence rates, postoperative complications), time from admission to first procedure, and resource utilization (total hospitalization charges and costs, and length of hospitalization). Statistical analyses were performed to determine frequencies, odds ratios, and significance. Statistical significance was set at p< 0.05. We controlled for the above-defined dependent variables in the logistic regression analysis.

**Results:**

Primary payer status was not associated with inpatient mortality in patients with HS (*p*=0.092). From 2017-2019, there were a total of five (0.04%) of in-patient mortalities in patients with HS, with all five being insured by Medicaid and occurring in 2017. Compared with private insurance, Medicare was associated with significantly lower incidences of wound dehiscence (aOR=0.21 (0.06-0.70); *p*=0.011) and longer time from admission to first procedure (mean days=1.85 vs 1.23; *p*=0.01). Medicaid was associated with significantly lower incidences of infection (aOR=0.21 (0.08-0.58); *p*=0.002) and longer time from admission to first procedure (mean days=1.73 vs 1.23; *p*=0.024). The only difference observed in the uninsured, when compared to privately insured, was significantly lower total costs ($10601.78 vs $13658.37; *p*=0.030).

**Conclusions:**

Our study demonstrates that in patients with HS, inpatient morbidity factors are overall better in those who are publicly insured over those privately insured. However, publicly insured patients face a longer wait to first procedure from admission over those privately insured.

**Applicability of Research to Practice:**

By raising awareness in treatment outcomes and resource utilization for patients with HS in inpatient settings, the medical team caring for these patients can begin to identify reasons for the disparities shown by insurance status.

